# Negative Affectivity, Authoritarianism, and Anxiety of Infection Explain Early Maladjusted Behavior During the COVID-19 Outbreak

**DOI:** 10.3389/fpsyg.2021.583883

**Published:** 2021-02-25

**Authors:** Vincenzo Bochicchio, Adam Winsler, Stefano Pagliaro, Maria Giuseppina Pacilli, Pasquale Dolce, Cristiano Scandurra

**Affiliations:** ^1^Department of Humanities, University of Calabria, Arcavacata di Rende, Italy; ^2^Department of Psychology, George Mason University, Fairfax, VA, United States; ^3^Department of Neurosciences, Imaging, and Clinical Sciences, University of Chieti-Pescara “G. D’Annunzio”, Chieti, Italy; ^4^Department of Political Sciences, University of Perugia, Perugia, Italy; ^5^Department of Public Health, University of Naples Federico II, Naples, Italy; ^6^Department of Neuroscience, Reproductive Sciences, and Dentistry, University of Naples Federico II, Naples, Italy

**Keywords:** COVID-19 outbreak, negative affectivity, right-wing authoritarianism, anxiety of infection, maladjusted behavior, pandemic

## Abstract

During the first phase of the COVID-19 outbreak, Italy experienced problems of public order and maladjusted behavior. This study assessed the role of negative affectivity, right-wing authoritarianism, and anxiety of COVID-19 infection in explaining a variety of the maladjusted behaviors (i.e., “China-phobic” discrimination, panic buying) observed with an Italian sample. Specifically, we examined the effect of Negative Affectivity and Right-Wing Authoritarianism on maladjusted behaviors, and the moderating role of anxiety of infection. Seven hundred and fifty-seven Italian participants completed an online survey between March 3rd to the 7th 2020, which was immediately before the lockdown. A moderated-mediation model was tested using a structural equation modeling approach. Results indicated that both Negative Affectivity and Right-Wing Authoritarianism were positively associated with COVID-19-related maladjusted behavior, and that Right-Wing Authoritarianism mediated the relationship between Negative Affectivity and maladjusted behavior. Furthermore, the effect of Right-Wing Authoritarianism on maladjusted behavior was greater for those with high anxiety of infection, and the indirect effect of Negative Affectivity on maladjusted behavior through Right-Wing Authoritarianism was moderated by infection anxiety. Findings highlight potential psychological paths that may inform communication strategies and public health initiatives aimed at promoting healthy behavior during an outbreak.

## Introduction

The novel coronavirus (COVID-19) outbreak was declared a public health emergency by the World Health Organization (WHO) on January 30th 2020, and quickly became the most significant, devastating, and challenging pandemic the world has experienced in recent history. Although initially the focus was on China, as the virus began to spread throughout the world, Europe (most notably Italy and Spain) became the hot spot for the virus and the focus of global attention before the outbreak went on (and continues) to devastate the United States and other countries worldwide. Italy, in particular, is a critical context for understanding human behavior in the early stages of a pandemic because Italy was the first country, after China, to have to deal with the virus on such a large scale with very limited information available at the time about the virus and about pandemics in general.

Given that there is currently neither definitive treatment for the disease nor a vaccine for preventing the infection, the only means currently available for preventing and limiting the COVID-19’s wider spread involves understanding and modifying human behavior (i.e., social distancing, frequent hand washing, mask wearing, quarantine). For this reason, the governments of most countries have adopted severe restrictions, imposing restrictive mass quarantine and stopping industrial, travel, and commercial activities. Because such preventive measures against the COVID-19 outbreak are psychological/behavioral rather than pharmacological, it is extremely important that people comply with the indications delivered from public health organizations and governments and avoid engaging in maladjusted or antisocial behavior that can cause problems of public order. In Italy, the context of our study, the early stage of the outbreak was unfortunately characterized by conflicting information about the nature of infection, and unclear and often contradictory instructions and suggestions were given to people to contain the outbreak by local and national government officials (Billeci, 2020).

Indeed, the early phase of the outbreak in Italy was sadly characterized by problems of public order and/or a variety of maladjusted behaviors in the population, for instance widespread “China-phobic” discrimination including verbal and physical aggression, and cases of panic buying to hoard domestic essential goods. The current study evaluated the role of relevant individual and social variables (i.e., personality, authoritarianism, and infection anxiety) in predicting such maladaptive behavior. Italy represents a unique and peculiar context for this assessment, because it was the first Western country that was hit by the outbreak. To our knowledge, this is the first study assessing the psychological and behavioral impact of the COVID-19 outbreak in the Italian population in the early stages of the public health emergency. In the following paragraphs, we provide a brief description of the evolution of the COVID-19 outbreak in Italy, focusing on the first stages of the infection. Then, we review the literature on the constructs hypothesized here to be related to maladaptive behavior in this context.

The evolution of the outbreak in Italy can be divided into the following phases:

**Phase 1.** The “Chinese” outbreak: From December 2019 to February 17th 2020. In this phase, there were no certified cases of infection in Italy, except for a couple of Chinese tourists, who were hospitalized in isolation in Rome on January 29th. The outbreak was perceived (and even communicated by most politicians) as a Chinese problem. The government decided to block all flights to and from China on January 30th, but no restrictions addressed to the Italian population were established. Italian public health institutions did not give specific instructions as to prevention of the infection.

**Phase 2.** The beginning of the outbreak in Italy: From February 18th to March 9th 2020. This phase began with the certification of the first Italian case of infection, which occurred in a small city of Lombardy (Codogno) on February 18th. In the days to follow, other cases of infections in Italian citizens were noted. The reactions of central and local government were uneven, unclear, and contradictory in some circumstances. Some cities and provinces of Lombardy and northern Italy were isolated, and a local lockdown was declared, but no general restrictions were established for the rest of the country. Even in the case of Lombardy, the center of infections in Italy, the situation was unclear. Indeed, while some cities and provinces were in lockdown, regional government, politics, influencers, and newspapers suggested not to stop social and economic activities in the regional county seat (i.e., Milan), and the hashtag #Milanononsiferma (Milan does not stop) was a top trend in social media. Spots encouraging people to lead “a normal life,” without restrictions, were repeatedly diffused into TV and social media outlets starting February 27th, including videos saying that self-imposing quarantine, avoiding traveling, and limiting one’s social life should NOT be done since these were irrational behaviors induced by fear and anxiety and would be detrimental for the economy (the spot for #Milanononsiferma is available at this URL^1^). The whole Lombardy region, and some other provinces of northern Italy, were then suddenly declared “red zones” with a complete lockdown beginning on March 8th, and a lockdown for the whole country was declared on March 9th.

In this phase, scientific communication was also characterized by divergent and partly contradictory information. In some cases, COVID-19 was described as “something more than a flu,” indicating older people as the only population at risk, and similar remarks occurred in the United States with Donald Trump’s public communications as well (Brooks, 2020). During this phase, public health organizations did not give coherent instructions, for instance, the Italian Ministry of Health suggested people should wash hands frequently, but declared face masks useless and in some cases dangerous (Billeci, 2020); or suggesting to maintain a safe distance from others, but declaring that people can continue to travel for work or social necessities. Some dangerous and unhelpful maladjusted behaviors were noticed within the Italian population during this phase as well such as (1) widespread “China-phobic” discriminatory actions, such as avoidance of Chinese people and their shops, and many cases of verbal and physical assaults on Chinese people (Di Fraia, 2020; Gorlani, 2020), and (2) the hoarding and stockpiling of domestic essential goods (Capovilla, 2020; Vazzana, 2020) creating serious supply problems, despite public authority’s requests to avoid such behavior. It is precisely during this second phase of the outbreak in Italy (March 3 to 7, 2020) when we collected survey data for the present study.

**Phase 3.** Italy in lockdown: from March 9th to May 18th 2020. Starting from the governmental decree of March 9th that imposed a lockdown for the whole country, several other decrees imposed progressively more and more social and commercial limitations. The government and the public health institutions provided clear, unambiguous, and strict indications in terms of personal hygiene, social distancing, freedom of movement, and productive/commercial activities (all decrees adopted by the Italian government are available at this URL^2^). No relevant cases of public disorder have been registered, except limited and individual episodes of lockdown infringement.

Our research was conducted during Phase 2 – characterized by contradictory indications from leaders and frequent problems of public order. The survey opened on March 3rd and ended on March 7th 2020 (after the lockdown in certain northern Italian provinces and right before the national lockdown). It is plausible to hypothesize that the lack of clarity and coherence regarding the evolution of the outbreak increased levels of state-anxiety in the Italian population which, in turn, increased the risk of adopting maladjusted behaviors. It is also plausible that, in such a confusing situation, some individuals might have personally wished for a more authoritarian, ordered, determined, and unambiguous public intervention and, thus, maladjusted behavior might be associated with specific personality traits (e.g., negativity affectivity) and state factors (i.e., anxiety of infection) of the individual.

Naturally, defining what is “adjusted” or “maladjusted” behavior in a context completely new and uncharted such as the current global pandemic is not easy, in particular because in this case, it is necessary to counterbalance two equally legitimate perspectives: an individual perspective (i.e., desire to protect individual health and wellbeing) and a social and public perspective (i.e., to maintain social cohesion, contain the virus, guarantee equal opportunities of access to primary goods and services, and avoid panic spreading in the population). Such a dichotomy between individual/selfish tendencies on the one hand, and collective and social motivations on the other, has been highlighted in many research fields, including intragroup regulation (Tyler and Blader, 2003; Ellemers et al., 2013; Ellemers, 2017) and organizational behavior (Organ, 1988; Ashforth and Mael, 1989). Researchers highlight that in many cases, individuals are inclined to reduce selfishness in favor of group-based behaviors, even showing discretionary (vs. mandatory) pro-group tendencies (Podsakoff et al., 1990; Blader and Tyler, 2009), in particular when they strongly identify with their in-group (Tajfel and Turner, 1979).

A useful construct for attempting a definition of adjusted behavior is Organizational Citizenship Behavior (OCB) theory (Smith et al., 1983; Organ, 1988; Podsakoff et al., 1990). The construct of OCB was originally developed in industrial/organizational psychology to indicate a complex set of discretionary behaviors and attitudes that an individual can display within an organization, contributing to its functioning (e.g., altruism, compliance with general rules and expectations, courtesy, conscientiousness, loyalty, and civic virtue; Hoffman et al., 2007). Williams and Anderson (1991) distinguish two distinct types of OCB: Organizational Citizenship Behavior-Organization, which includes “behaviors that benefit the organization in general”; and Organizational Citizenship Behavior-Individual, which includes “behaviors that immediately benefit specific individuals” (pp. 601–602).

Although developed within organizational psychology, in the attempt to define and comprehend the features of adjusted and maladjusted behavior and attitudes during a global pandemic, OCB-Individual and OCB-Organization have heuristic value, because they provide an instrument within the pandemic that conceives of behavior oriented both toward individuals and local/national organizations. Following this heuristic attempt at conceptualization, we adapt the OCB-Organization construct to the present situation of a pandemic, defining as “adjusted” behaviors aimed at avoiding negative consequences for managing the pandemic or causing more problems of public order in an already critical situation. By contrast, a “maladjusted” behavior could be defined as a behavior by which individuals intend to preserve or protect themselves, without regard for the negative consequences that such behavior can have on social groups, economy, and society, and more generally on the public organization facing the pandemic. Continuing with the organizational metaphor, such behaviors can also be assimilated as Counterproductive Work Behaviors (Spector and Fox, 2005), selfish behaviors that can be detrimental for the organization in which an individual works. Interestingly for the present purpose, researchers consistently show that in organizational contexts, OCB and Counterproductive Work Behaviors are related to how strongly the individual identifies with the organization in opposite ways. Higher organizational identification is positively associated with OCB and negatively associated with Counterproductive Work Behaviors (Pagliaro et al., 2018).

Remembering the period when we collected data, right before the full lockdown, an emblematic case of such maladjusted behavior would be the antisocial behavior that took place at supermarkets while trying to stock up and hoard primary goods. This kind of behavior creates problems of public order and causes the temporary unavailability of important goods, even though governments claimed that supermarkets would never be closed and goods would always be available. Another example of questionable behavior observed at the time was self-imposition of a strict quarantine and avoidance of going to work even when the government declared that such work was essential and needed for the country’s economy, especially in the case of health and food production sectors.

Similarly, we adapt the OCB-Individual construct to the present situation of the pandemic, defining as “adjusted” behaviors characterized by altruism, public virtue, friendship, and activities that help disadvantaged people. By contrast, a “maladjusted” behavior could be defined as a behavior by which individuals, with the goal of protecting or preserving themselves, express attitudes and behaviors that implicitly or explicitly harm individuals belonging to disadvantaged and/or minority groups. An emblematic case of this kind of maladjusted behavior during the first phase of the pandemic in Italy was avoiding Chinese people (or Asian individuals more generally), boycotting their places of business, and harassing them verbally and physically.

Maladjusted behavior seems to be strongly associated with Negative Affectivity (NA) as a personality trait. A recent meta-analysis on OCB suggested a relationship between NA (i.e., an individual’s disposition to experience feelings such as anger or trait anxiety, have and labile emotional states, and engage in hostile interpersonal behavior; Watson et al., 1988) and facets of both OCB-Individual and OCB-Organization (Geiger et al., 2019). According to this perspective, NA would have a stronger relationship with OCB than state negative affect, and NA would have a stronger relationship with OCB-Organization than OCB-Individual (Geiger et al., 2019). This means that, at different levels, both trait and state anxiety can be considered predictors of OCB; specifically, people higher on NA may engage in less OCB. Following these indications, it is plausible to hypothesize that NA would increase the likelihood of adopting maladjusted behaviors during Phase 2 of the pandemic in Italy, particularly because it is likely that in this phase, people experienced high levels of state anxiety due to the general uncertainty and fear associated with the evolution of a pandemic and its effects on society and the population (Hirsh et al., 2012).

Exposure to threatening events perceived as disruptive for social cohesion and personal security can also affect individuals’ subjective levels of authoritarianism (Feldman and Stenner, 1997). Right-Wing Authoritarianism (RWA; Altemeyer, 1981) has been the object of intense research in recent decades, and some theoretical frameworks that posit causes, sub-constructs, antecedents, and eliciting factors of RWA have been proposed (Sibley and Duckitt, 2008; Duckitt et al., 2010). Here we intend Authoritarianism to be more of a state condition – a set of beliefs and behavior that are sensitive to social events and therefore susceptible to change – rather than a stable trait condition, but it is important to highlight a long tradition of studies in the field that tend to consider Authoritarianism as a highly stable personality trait, relatively unaffected by the individual’s unique experiences (Altemeyer, 1996; Ludeke and Krueger, 2013; Adorno et al., 2019). Among those who consider that the levels of Authoritarianism are sensitive to social events, an interesting theoretical model was proposed by Jugert and Duckitt (2009). The authors suggest that subjective levels of RWA can be caused by what they call collective security motivation, that is “the motivational goal or value that the collective one identifies with and lives in should be safe, secure, predictable, harmonious, stable, cohesive, and orderly” (p. 696). In addition, Jugert and Duckitt (2009) argue that one’s level of collective security motivation provides a measure of sensitivity to threats of social disruption and danger, which leads to a personal desire for social order, and a need for stability, predictability, and social control. Thus, collective security motivation can explain the requests for order, social control, and stability seen in people with high levels of RWA. This framework sees RWA as a subjective variable, highly sensitive to particular disruptive events (as observed with the World Trade Center attacks on September 11th 2001; Nagoshi et al., 2007). In this sense, it is plausible that a critical event like the COVID-19 epidemic – by eliciting anxiety and fear – would increase RWA. Much research provides evidence for a mediating role of RWA, for instance previous studies found that RWA mediates the relationship between religious fundamentalism and attitudes toward specific minority groups (Johnson et al., 2012), between religious fundamentalism and racism (Johnson et al., 2011), and between dangerous world beliefs (such as “Any day now chaos and anarchy could erupt around us” and “There are many dangerous people in our society who will attack someone out of pure meanness, for no reason at all”; Altemeyer, 1988) and attitudes toward human rights/civil liberties (Crowson, 2009). It is, thus, plausible that RWA would mediate the relationship between a stable independent variable (e.g., NA) and maladjusted behavior.

In addition, since perceived societal fear and anxiety would elicit an increase in RWA (Manzi et al., 2015), it is also plausible to hypothesize that perceived anxiety of COVID-19 infection would act as a moderating variable between NA, RWA, and maladjusted behavior. Indeed, high levels of reactivity and arousal of state-anxiety seem to lead to an impairment in decision-making processes, thus an increase in maladjusted behavior (Luhmann et al., 2011). In fact, anxiety may alter the process through which people make decisions, interfering with people’s ability to process information because of cognitive biases (Hartley and Phelps, 2012), and this may lead to an impairment in goal-directed actions (Alvares et al., 2014). For these reasons, it is plausible to hypothesize that infection anxiety may increase the probability that people high in trait anxiety engage in “maladjusted” behavior, that is, actions and behaviors that can have negative consequences on other individuals, social groups, economy, and society, and more generally on the public organization aimed at facing the pandemic.

**FIGURE 1:**
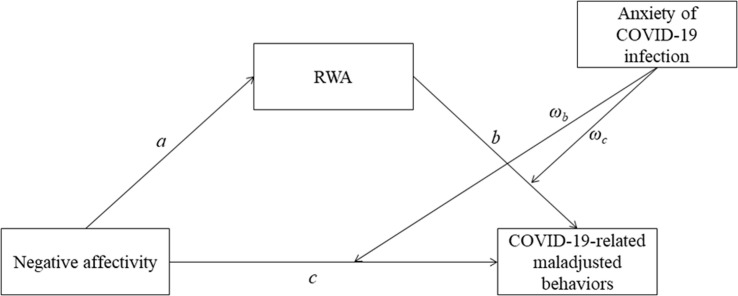
The hypothesized moderated-mediation model. RWA, Right-Wing Authoritarianism.

The current study assessed the role of NA, RWA, and infection anxiety in explaining maladjusted behaviors observed by Italian individuals during the second phase of the COVID-19 outbreak in Italy. Specifically, on the basis of OCB theory (Smith et al., 1983; Organ, 1988; Podsakoff et al., 1990) and the collective security motivation framework (Jugert and Duckitt, 2009), we firstly hypothesized that NA would be positively associated with RWA, that both NA and RWA would be positively associated with COVID-19-related maladjusted behaviors, and that RWA would also act as a mediator between NA and COVID-19-related maladjusted behaviors (Hypothesis 1). Additionally, on the basis of the relationship between anxiety and maladjusted behavior (Luhmann et al., 2011) and with the aim of assessing the potential risk factor of infection anxiety, we secondly hypothesized that higher levels of infection anxiety would increase the effect of both NA and RWA on maladjusted behavior, thereby moderating these relationships (Hypothesis 2). Furthermore, we hypothesized that higher levels of infection anxiety would increase the effect that NA would have on COVID-19-related maladjusted behaviors through the mediating action of RWA (Hypothesis 3). The hypothesized moderated-mediation model is depicted in Figure 1.

## Materials and Methods

### Procedures

The current study used a cross-sectional online survey. The survey was launched on social media (e.g., Facebook) between March 3rd and March 7th 2020 and participants were recruited through a snowball sampling recruitment procedure, encouraging them to spread the survey to others. In spreading the survey, great attention was given to cover all Italian regions, by posting the survey on public online regional groups with large number of members. By clicking on the link provided, participants were directed to the first page of the survey containing the informed consent of the study, objectives, benefits, risks, and information about the researchers. Participants were informed about the anonymity of the survey, as well as the time needed to complete it (approximately 15 min). At the end of the survey, participants were informed about the possibility of receiving a short report on results and were invited to send their emails to the Principal Investigator (PI) if they desired the report.

Privacy was guaranteed in accordance with the EU General Data Protection Regulation 2016/679, on whose basis data were protected by a secure gateway accessible only to the PI, who removed all IP addresses before sharing the dataset with other researchers. The study was designed to respect all the principles of the Declaration of Helsinki on Ethical Principles for Medical Research Involving Human Subjects and was approved by the ethical committee of the University of Calabria (protocol number 8104).

### Participants

Participants of the current study were recruited just before the lockdown of the country, when only some Northern regions were at high risk. Inclusion criteria were: (1) being at least 18 years old, the Italian age of consent, and (2) living in Italy assessed via self-report. A total of 757 Italian participants completed the survey (183 males, 571 females, and 3 transgender/other). Participants ranged in age from 18 to 78 years old (*M* = 34.96, *SD* = 11.88). Overall, 18.8% (*n* = 142) of the sample live in a zone declared at risk, 71.6% (*n* = 542) was highly educated (college degree or above), and only 3.7% (*n* = 28) personally knew an infected person. Finally, most of the sample (*n* = 381; 50.3%) said that they primarily got COVID-19 information from official websites (e.g., Ministry of Health), 14.9% (*n* = 113) from unofficial websites (e.g., blogs), 23.9% (*n* = 181) from TV, 7.1% (*n* = 54) from newspapers, and 3.7% (*n* = 28) from other sources (e.g., Facebook).

### Measures

#### Socio-Demographic Characteristics

Socio-demographic variables used in the current study included age, gender identity (women, men, and other), level of education (1 = high school or less; 2 = college or more), Italian regions (*n* = 21) in which participants lived, and main channels used to be informed on COVID-19 (e.g., official websites, unofficial websites, TV, etc.). Furthermore, we asked participants if they personally knew someone who had been infected with the COVID-19 and if they lived in a zone declared at risk, specifying which area.

#### Negative Affectivity

Negative Affectivity was measured through the subscale of the Personality Inventory for DSM-5 Brief Form (PID-5-BF; Krueger et al., 2011; Italian version by Fossati et al., 2013), a self-report questionnaire consisting of 25 items scored on a 4-point Likert scale, from “very false or often false” to “very true or often true.” Example items of the subscale are “I worry about almost everything” or “I get emotional easily, often for very little reason.” The score is calculated by dividing the raw score by the number of items, with higher scores reflecting more NA. Internal consistency reliability of the measure was 0.90 for the Italian normative sample (*N* = 1,544) (Fossati et al., 2013). The alpha coefficient in the current sample was 0.71. This scale has been used in several Italian studies (e.g., Granieri et al., 2017; Anzani et al., 2020).

#### Authoritarianism

Authoritarianism was assessed through the 10-item Italian version of the RWA scale (Altemeyer, 1996; Italian version by Roccato and Russo, 2015). An example item is “Our country needs a powerful leader, in order to destroy the radical and immoral currents prevailing in society today.” Items scored on a 4-point Likert scale, from “strongly disagree” to “strongly agree,” with higher scores indicating higher RWA. Internal consistency reliability of the measure was 0.83 for the Italian validation sample (*N* = 839) (Roccato and Russo, 2015). The alpha coefficient in the current sample of the measure was 0.72. This scale has been effectively used in several Italian studies (e.g., Manzi et al., 2017; Spaccatini et al., 2019).

#### Anxiety of COVID-19 Infection

Infection anxiety was measured through 20 items adapted by Wong et al. (2007) who conducted a similar study during the SARS epidemic in Hong Kong. We kept all items the same but just replaced “SARS” with “coronavirus,” and adapted some specific items to the Italian context (e.g., the item “I feel that it is difficult to control the SARS epidemic in such a dense city as Hong Kong” was adapted to “I feel that it is difficult to control the coronavirus epidemic in the most densely populated cities”). Participants were asked to think about their behaviors and emotions related to the COVID-19 outbreak in Italy and answered questions on a 4-point Likert scale ranging from “strongly disagree” to “strongly agree” (e.g., “I am afraid I have been infected with the coronavirus”). The alpha coefficient of the measure in the current sample was 0.89.

#### COVID-19-Related Maladjusted Behaviors

Maladjusted behaviors associated with COVID-19 were measured through 5 items created for this study. Participants were asked to answer questions on the frequency of different behaviors related to the COVID-19 outbreak during the past 2 weeks on a 4-point Likert scale ranging from “never” to “very often.” Specifically, participants were asked how much they (1) avoided Chinese people and stores (i.e., “In the past 2 weeks, I have avoided Chinese people and/or their stores”), (2) stockpiled food and goods (i.e., In the past 2 weeks, I have been stocking food and goods”), (3) limited their social life (i.e., “In the past 2 weeks, I have limited my outings and my social life”), (4) given up traveling (i.e., “In the past 2 weeks, I have given up traveling”), and (5) forced themselves to a quarantine (i.e., “In the past 2 weeks, I have imposed on myself a quarantine, remaining at home”).

At the time of the survey, such behaviors were not suggested by the local or national authorities and were instead considered maladjusted behaviors induced by unjustified anxiety and fear which was dangerous for the national economy and public order. Naturally, *post hoc* we can say that at least the last three items could be considered, in fact, adjusted behaviors, but at the time of the survey national and local governments, influencers and politics recommended strongly to avoid behaviors that could have been potentially detrimental for the economy, such as avoiding going at work or traveling, particularly in Milan and Lombardy. Spots for encouraging people to lead “a normal life” without restrictions were constantly shown on TV and social media starting from February 27th (the spots are available at these URL see text footnote 1; URL^3^). For these reasons, the original research design considered all these items as “maladjusted behaviors,” because during Phase 2 of the outbreak in Italy, the National Institute of Health and the Ministry of Health established, and politicians, influencers, TVs, and newspapers communicated, that all the behaviors indicated in the questionnaire were problematic, maladjusted, and induced by anxiety and fear. Indeed, a principal components analysis was performed, showing a one-factor solution which explained 51.61% of the variance and factor loadings ranging from 0.60 to 0.84. Correlations between the items ranged from 0.25 to 0.64 supporting their combination into one factor. The Cronbach’s coefficient of the scale was 0.75. Obviously, the situation changed dramatically during Phase 3 of the outbreak, when the last three behaviors were not simply suggested by the authorities, but strictly imposed. Therefore, in addition to analyzing the entire scale, we also decided to take into account potential differences, distinguishing between behaviors that were always considered maladjusted (i.e., items 1 and 2; “Always maladjusted behaviors”), and behaviors that were discouraged in the second phase, but imposed in the third phase of the outbreak (i.e., items 3, 4, and 5; “Maladjusted behaviors only during phase 2”).

### Statistical Analyses

Statistical analyses were performed using the R software environment, setting the level of significance at 0.05. Pearson’s correlation coefficients were used to estimate bivariate correlations between variables. As socio-demographic variables may influence COVID-19-related maladjusted behaviors, we adjusted the models with a variety of confounding variables, including age, gender (excluding the 3 transgender/other participants), educational level (≤High school vs. ≥College), personal knowledge of infected people, and living in a zone declared at risk. Specifically, as being older, being male, and living in areas at high risk of infection represent risk factors for higher severity and mortality (Jin et al., 2020; Jordan and Adab, 2020), it is plausible to hypothesize that such factors may influence both anxiety of infection and behavior. Similarly, it is also plausible to hypothesize that direct knowledge of infected people may increase both infection anxiety and maladjusted behaviors. Finally, because less educated people, and those with lower levels of COVID-19 knowledge (Zhong et al., 2020) may have higher levels of anxiety (Lei et al., 2020), education may be considered another confounding variable affecting both anxiety of infection and behavior.

Moderated mediation analysis was conducted to test the hypotheses of the study. Moderating and mediating effects were specified and tested according to the recommendations provided by Holmbeck (1997). The structural equation modeling approach was performed using weighted least squares estimation with robust standard errors and a minimum required sample size of at least 200 participants (Kline, 2015). All moderated mediation analyses were performed using the Lavaan R package (Rosseel, 2012).

First, a mediation model was fit, with NA as predictor, COVID-19-related maladjusted behaviors as the outcome, and RWA as the mediator. The outcome (i.e., COVID-19-related maladjusted behaviors) was specified in the model as a second-order factor underlying the two first-order factors (i.e., “Always maladjusted behaviors” and “Maladjusted behaviors only during phase 2”). Then, a moderated mediation model was performed, with infection anxiety as the moderator. The analyses were performed in two steps (see paths reported in Figure 1). First, we tested the main effects of NA (c) and RWA (a) on maladjusted behaviors, and the mediating role of RWA on the relationship between NA and maladjusted behaviors (a^∗^b) (Hypothesis 1). Second, we tested the moderating effect of infection anxiety on relationships between both NA (ωc) and RWA (ωb) and maladjusted behaviors (Hypothesis 2), as well as on the effect of NA on maladjusted behaviors through RWA (Hypothesis 3). To evaluate the full moderated mediation model and provide evidence of moderation of the mediation effect, we estimated the Index of Moderated Mediation (IMM). Finally, the total fit of the model was assessed through the following indices: chi square/degrees of freedom (χ2/df), root mean square error of approximation (RMSEA), standardized root mean square residual (SRMR), comparative fit index (CFI), and Tucker–Lewis index (TLI). Values of χ2/df < 2, RMSEA and SRMR < 0.08, and TLI and CFI > 0.95 are indicative of a good fit with the data (Kline, 2015).

## Results

### Descriptive Statistics and Bivariate Correlations

Means, SD, and bivariate correlations between NA, RWA, maladjusted behavior, and infection anxiety are shown in Table 1. The results showed that all variables correlated somewhat with each other. Specifically, NA positively correlated with RWA, infection anxiety, and maladjusted behaviors.

**TABLE 1:** Correlations between negative affectivity, RWA, anxiety of infection, and maladjusted behaviors.

***Scales***	**1**	**2**	**3**	**4**	**5**	**6**	**Mean**	***SD***
1. Negative Affectivity	−						1.08	0.65
2. RWA	0.10**	−					1.79	0.46
3. Anxiety of infection	0.25***	0.10**	−				2.25	0.54
4. Maladjusted behaviors (tot)	0.11**	0.18***	0.53***	−			1.68	0.66
5. Always maladjusted	10**	28***	0.35***	75***	−		1.47	0.68
6. Only during phase 2	0.09*	0.09*	0.52***	0.93***	0.46***	−	1.83	0.82

*p<.05, **p<.01, ***p<.001. RWA, Right-Wing Authoritarianism; SD, Standard Deviation.

### Direct and Indirect Associations Between NA, RWA, and Maladjusted Behaviors

**FIGURE 2:**
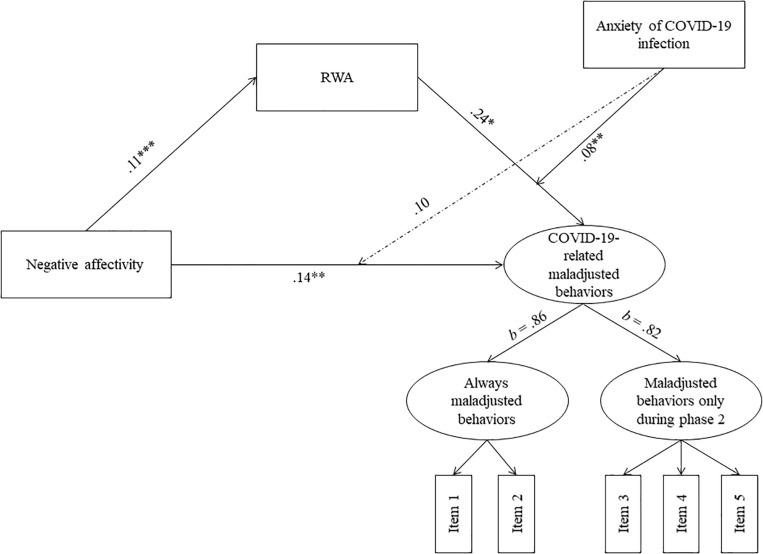
Results from the moderated-mediation model. Dashed lines represent non-significant paths. For simplicity, associations with control variables are omitted. RWA, Right-Wing Authoritarianism. ^∗^*p* < 0.05, ^∗∗^*p* < 0.01, and ^∗∗∗^*p* < 0.001.

As shown in Figure 2 and with respect to Hypothesis 1, results indicated that NA was positively associated with RWA, *a* = 0.11, *p* < 0.001, *95% CI* [0.05, 0.16] and that both NA, *c* = 0.14, *p* = 0.001, *95% CI* [0.05, 0.23], and RWA, *b* = 0.24, *p* = 0.04, *95% CI* [0.11, 0.37], were positively associated with COVID-19-related maladjusted behaviors. Furthermore, we found that RWA significantly and positively mediated the relationship between NA and COVID-19-related maladjusted behaviors, a^∗^*b* = 0.03, *p* = 0.007, *95% CI* [0.01, 0.04]. Specifically, RWA increases as NA heightens and, consequently, COVID-19-related maladjusted behaviors increase. Furthermore, coefficients of the two first-order variables measuring maladjusted behaviors were *b* = 0.86 and *b* = 0.82, respectively, indicating that the second order factor loaded very similarly to the first. These findings confirmed Hypothesis 1. Finally, none of the control variables had a statistically significant effect on the variables in the model.

### The Moderating Role of Infection Anxiety

With regard to Hypothesis 2, we found only a significant and positive interaction between RWA and infection anxiety on COVID-19-related maladjusted behaviors, *ω_*b*_* = 0.08, *p* = 0.001, *95% CI* [0.03, 0.13], indicating that the effect of RWA on maladjusted behaviors increases the more people feel anxiety about infection, partially confirming our hypothesis. By contrast, there was no evidence that infection anxiety moderated the relationship between NA and maladjusted behaviors, *ω_*c*_* = 0.10, *p* = 0.06, *95% CI* [−0.01, 0.20].

**FIGURE 3:**
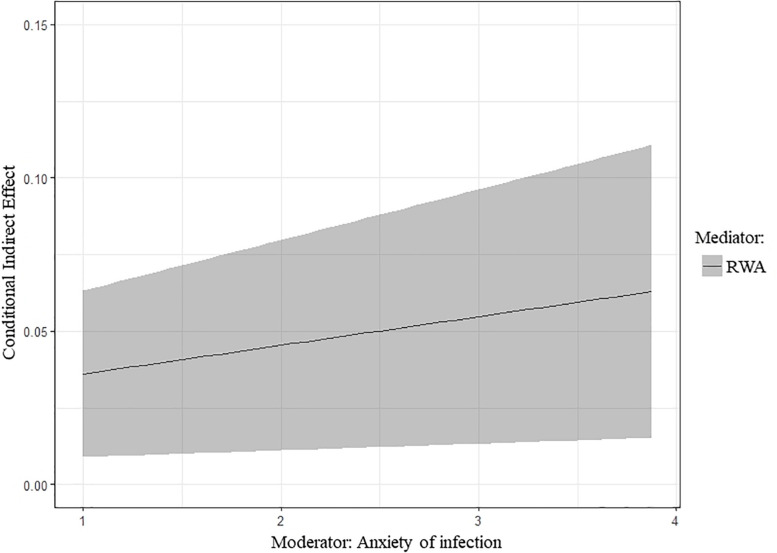
Conditional indirect effect, along with the 95% confidence intervals, of negative affectivity on COVID-19-related maladjusted behaviors through RWA as a function of anxiety of COVID-19 infection. RWA, Right-Wing Authoritarianism.

With regards to Hypothesis 3, results indicated that the indirect effect of NA on COVID-19-related maladjusted behaviors mediated by RWA was significantly moderated by infection anxiety, *IMM* = 0.01, *p* = 0.006, *95% CI* [0.003, 0.020], confirming our hypothesis. Specifically, the indirect effect increases as anxiety increases, confirming that infection anxiety might be a risk factor increasing the negative effects that NA has on maladjusted behaviors (Figure 3). Measures of model fit were as follows: *χ2*/*df* = 94/41, RMSEA = 0.04, SRMR = 0.04, CFI = 0.95, TLI = 0.93.

## Discussion

The current study was aimed at assessing the role of NA, RWA, and anxiety of infection in explaining maladjusted behavior in the early phase of the COVID-19 outbreak in Italy. To the best of our knowledge, this is the only Italian study assessing these relationships in the early phases of the outbreak in Italy, that is, before the national lockdown. In the original design of the research, we took into consideration five behaviors as maladjusted, but looking at the evolution of the Italian public health policies in Phase 3 of the outbreak, we also considered potential differences in these behaviors. Our results suggested that, at the time of the survey, no differences between such behaviors were statically supported, as the loadings representing the relationships between the second order factor with the two first order factors were very similar. This might mean that in Phase 2 of the outbreak in Italy all these behaviors were part of a unique latent factor indicated maladjusted behavior. For these reasons, in this discussion, we refer to “COVID-19-related maladjusted behaviors” including all five behaviors included in our COVID-19-related maladjusted behaviors questionnaire.

Specifically, in support of our first hypothesis, we found that both NA and RWA were associated with COVID-19-related maladjusted behaviors, and that RWA mediated the relationship between NA and maladjusted behaviors. These results are partially supported by the literature. Following the interpretation of COVID-19-related maladjusted behaviors as behaviors with low levels of OCB, we can assert that our results confirmed previous studies finding a strong relation between NA and various facets of OCB, with NA increasing the likelihood of engaging in maladjusted behavior (behaviors characterized by low levels of OCB; Jain et al., 2012; Geiger et al., 2019). This means that, during phase 2 of the COVID-19 outbreak in Italy, people who experienced NA were more likely to adopt the maladjusted behaviors assessed in our survey.

In this scenario, authoritarianism seemed to play a mediating role between NA and maladjusted behaviors, and this could be better understood considering the specific characteristics of the moment during which the survey was launched. Phase 2 of the COVID-19 outbreak in Italy was characterized by uneven, unclear, and contradictory information about the infection and even more unclear indications given by the central government, local administrations, and public health institutions. Summarizing, it was a phase of deep “disorder” and uncertainty in relation to what people needed to do in order to avoid the spread of the infection. Since previous studies found that NA is strongly characterized by a high intolerance of uncertainty (Norton and Mehta, 2007), the mediating role of RWA may be interpreted as an implicit need of people with negativity affectivity to have order and social control, induced by the perception and threat that the situation was uncertain and out of control. In turn, as threat impairs perceived control, RWA increases as a function of low control (Fritsche et al., 2011; Manzi et al., 2015) and is a reaction to this threat from external danger (Onraet et al., 2013). This finding could be interpreted as an implicit strategy for coping with the threat that the social context was dangerously “out of control” without clear and prescriptive rules of behavior, because part of RWA is a desire for a highly structured and controlled societal system and for an authority that imposes structure and order. In other words, desiring more social order and control may represent a coping strategy for managing the perception that the living context is dangerously insecure and out of control.

This interpretation is also consistent with the framework proposed by Jugert and Duckitt (2009), who see a direct cause of authoritarianism being collective security motivation. It is therefore plausible that during Phase 2 of the outbreak in Italy, RWA in people particularly sensitive to anxiety, fear, and intolerance of uncertainty is expressed as an implicit need for clearer management of the outbreak and enhanced personal desires for order and control in the societal system. The paradox is that the personal need for order and societal control, shown by the mediating role of RWA, ends up increasing the likelihood of acting in maladjusted ways that, in turn, may also provoke additional problems of public order.

Perhaps maladjusted behaviors expressing low levels of OCB-Individual (i.e., avoiding Chinese people and their shops or harassing them verbally or physically) are due to the hostility and prejudice that RWA generally produces toward groups perceived as “dangerous” (Duckitt and Sibley, 2007). But what about maladjusted behaviors that display low levels of OCB-Organization? Following our results, we draw the conclusion that an implicit need and request for order, social security, and stability, particularly in people with high NA, increases the likelihood to engage in behaviors that, in a critical situation like a pandemic, contribute to increased social disorder and confusion. Although it may seem bizarre or paradoxical, this finding seems to be consistent with some features of the “authoritarian specter” described by Altemeyer (1996). This refers to the combination of numerous cognitive failings and contradictory ideas, frequent false inferences in arguments, contradictory principles, and strong cognitive compartmentalization that produce a double standard in evaluation and decision making. Altemeyer (1996) concludes that when people fall in the “authoritarian specter,” they “use so many double standards that their behavior shows relatively little fairness and integrity. They may present themselves as highly principled people, but their principles shift quickly to justify whatever they happen to want – a shift they probably never notice.” (pp. 144–145).

Based on this definition, it is plausible that the clear dissonance between a need (i.e., social order and stability) and the outcomes of the behaviors associated with such a need (i.e., maladjusted behaviors low in OCB and leading to problems of public order) represent a case of “blindness” within the authoritarian specter.

In support of our second and third hypothesis, we found that the effect of RWA on maladjusted behavior increased if people felt anxiety about COVID-19 infection. By contrast, we did not find evidence for the moderating role of infection anxiety on the relationship between NA and maladjusted behavior. Furthermore, we found that the indirect effect of NA on maladjusted behavior through RWA was moderated by infection anxiety, supporting the hypothesized moderated-mediation model. Specifically, NA would increase the likelihood of adopting maladjusted behavior through the action of RWA in participants with high levels of anxiety of infection, but not in those with low levels of anxiety, thus highlighting that infection anxiety would be a risk factor for the adoption of maladjusted behaviors.

Consistently with the literature, people high in trait-anxiety are more reactive to state-anxiety (Glotzbach-Schoon et al., 2013), and this means that they are overly sensitive to threatening and dangerous events or situations, particularly when these events are characterized by high levels of uncertainty and unpredictability, and this is the reason why people suffering from anxiety disorders express high vulnerability to unpredictability and uncertainty (Mineka and Zinbarg, 2006). This high level of reactivity and arousal increases, in turn, the propensity to make hurried decisions that avoid uncertain or risky consequences, and this could lead to impairment in decision-making processes (Luhmann et al., 2011), and ultimately to maladjusted behavior. Our results are consistent with these findings, particularly because many items of the scale we used for assessing infection anxiety were related to fear for the future. This means that anxiety of infection would be particularly due to the infection’s unpredictability and unclear developmental course, and this would have to do with the individuals’ perception of the infection, particularly of it being (or not) “under control” or predictable in its development.

Finally, it is plausible that, in Phase 2 of the outbreak in Italy, local and central governments gave the impression that the situation was out of control, and that this increased anxiety in the population (Gasparro et al., 2020; Maldonato et al., 2020). Given that anxiety of infection moderated the relationships between NA, RWA, and maladjusted behaviors, to decrease the likelihood that a significant part of the population would act in a maladjusted manner creating problems of public order, perhaps interventions should focus on reducing levels of anxiety in the population. Infection anxiety is likely more malleable through intervention than both RWA and NA. This hypothesis is indirectly supported by the evidence that infection anxiety did not moderate the relationship between NA and maladjusted behaviors. Indeed, this suggests that state anxiety buffers the effect of NA on maladjusted behavior only in the presence of RWA when social security motivation, need for social control, and requests for prescriptive rules for behavior are present.

Our findings should be considered with respect to several limitations. First, the single point in time, cross-sectional nature of the study prevented us to make inferences about temporality and causality within the explored relationships. Future studies should consider implementing longitudinal designs to discern cause-effect relationships between NA, RWA, anxiety of infection, and maladjusted behavior. Second, although sample size was large, it was not representative of the Italian population, and this prevents us from generalizing our findings to the whole Italian context. Similarly, being that our sample is constituted by only Italian individuals, our findings must be interpreted within that cultural context. Furthermore, the sample is unbalanced in terms of gender and educational level, with higher rates of women and highly educated participants. However, these variables were considered in the model as potential confounders, adjusting the direct and indirect effects for these variables, even though their effects were not statistically significant. Notwithstanding, future studies should do better to recruit more balanced samples. Third, at the time of our survey, no specific measure on COVID-19 infection anxiety existed yet. It was not until March 27th, 2020 that the Fear of COVID-19 Scale was published (Ahorsu et al., 2020). Fourth, the spread of COVID-19 was so rapid in Italy and, subsequently, in Europe and the United States, that it was very difficult to classify certain types of behavior as between “correct” or “maladjusted,” because that depends on the information present at the time and what people are told by their governments, and those changed rapidly. To this end, we relied on a heuristic model from Industrial/Organizational psychology. Indeed, some of the behaviors that were considered maladjusted in this study were subsequently recommended by the government (i.e., quarantine). However, this represents at the time the special nature and contribution of our work, since at the time of data collection, these behaviors were strongly discouraged by the institutions.

## Conclusion and Implications for Public Policies

Despite limitations, our study may have some implications for public policy. According to our findings, maladjusted behaviors and problems of public order in Phase 2 of the COVID-19 outbreak in Italy were likely due to the perception that public management of the epidemic was out of control by central and local governments, as well as by public health institutions. In that phase, instructions and information on the individual and public management of the epidemic were uneven, unclear, and often contradictory, and this certainly increased the need and the personal desires for more order and social control in people experiencing anxiety and intolerance of uncertainty. This “authoritarian” need, induced by collective security motivation, would probably explain a set of behaviors characterized by low levels of civic virtue, courtesy, conscientiousness, loyalty, and altruism, that contributed to problems of public order. In this venue, it is meaningful that since the central government, local administrations, and public health institutions started to give clear, coordinated, univocal, and consistent instructions to the population, problems of public order no longer occurred, although restrictions were heavy and highly stressful. The moderating role of anxiety of infection is crucial in this scenario, as high levels of state-anxiety in particularly vulnerable people due to trait-anxiety seemed to lead to impairment in decision making processes, fostering the adoption of maladjusted behavior as a paradoxical expression of the need for social order and control.

Our findings may have significant implications for the management of social order and security in a case of serious crises such as an outbreak, as they seem to suggest that a functional way to contain maladjusted behavior in the population could be to take advantage of the mediating role that RWA seems to perform between NA and maladjusted behaviors, and of the moderating role that anxiety of infection seems to perform between the increased levels of RWA and maladjusted behavior.

To this end, since anxiety of the outbreak is particularly due to the perception of its unpredictability, effective public communication should be clear, well-defined, with unambiguous instructions. Further, such instructions should also be paired with clear data and verifiable predictions and end goals showing, for example, that if we do these restrictions for X amount of weeks, it should lead to X amount of reduction in virus spread, and if we get to our goal of X infections, we can start to lift certain restrictions, etc. Indeed, communicating the necessity of strict and heavy restrictions aimed at controlling the outbreak and the possibility to verify the benefits of such restrictions in a defined temporal range could have the effect of containing anxiety due to the unpredictability of the virus.

Furthermore, the mediating role of RWA seems to suggest that people with NA would need to perceive that the government and the authorities of public health can control the outbreak by prescribing clear and unambiguous behavioral indications (i.e., what to do exactly and what not to do), and this would probably buffer the intolerance for uncertainty. Our findings and the recent history of the COVID-19 outbreak in Italy seem therefore to suggest that maladjusted behaviors engaged in by a subset of the population are partly due to inconsistent indications and to the incoherent information provided by governments and leaders and public health institutions early in an outbreak, and therefore clear, unambiguous, and predictable public communication might reduce maladjusted behaviors, promote both social cohesion and better management of infection.

These findings may be important as, until a vaccine or a therapy for the infection becomes available, new waves of infections are quite likely, and in those occasions, governments and public health institutions should avoid the mistakes made in Italy (and currently in the United States) in terms of early public communication in order to promote social cohesion and maintain public order in a population. Indeed, currently in the United States, there has been no clear, univocal, strong Coronavirus strategy at the federal level with only mixed and contradictory messages coming from leadership. Instead there is massive variation from state to state in terms of social distancing restrictions and both the timing and duration of lockdowns, which in turn is associated with differential infection rates across communities and general failure to contain the virus (Fox et al., 2020).

To conclude, in Italy once the strict lockdown went into place, problems of public order and maladjusted behaviors rapidly and dramatically decreased. We hypothesize that the severity of restrictions imposed on a population can be well endured if an effective public communication strategy is able to contain anxiety in the population. The SARS outbreak in 2002–2003 showed that an “authoritarian response” for containing viral infections, i.e., heavy restrictions and limitation of personal freedoms, severe checks by law enforcement agencies, and strict imposition of social control, is very effective for avoiding widespread infection. This represents the so called “authoritarian advantage” of authoritarian regimes such as China (Schwartz, 2012). Indeed, in Italy and other European countries, the “authoritarian response” (i.e., severe mass quarantine, strict checks by law enforcement agencies, closure of all industrial and commercial activities except those related to food and healthcare goods), produced an impressive decrease in cases of infection in less than 2 months. In Italy, for instance, the peak of new daily cases of infection was reached on March 21st, a day in which 6,557 new cases of infection occurred, while after 2 months of mass quarantine, on May 22nd, only 652 new cases of infection were registered, which is 90% less.

Nevertheless, an “authoritarian response” could represent a political problem for a democratic regime, and especially for a population that usually benefits of a wide range of personal freedoms (such as the United States). Our study suggests that a democratic country can endure a temporary “authoritarian response” to a health crisis if governments are able to “enhance public trust by developing mechanisms to increase government transparency and interaction with the public” (Schwartz, 2012, p. 330), and this can be achieved by transparent, unambiguous, and predictable public communication. By contrast, ambiguous, uneven, unclear, and contradictory public communications can lead to maladjusted behaviors and problems of public order in a democratic regime.

## Data Availability Statement

The raw data supporting the conclusions of this article will be made available by the authors, without undue reservation.

## Ethics Statement

The study was designed to respect all the principles of the Declaration of Helsinki on Ethical Principles for Medical Research Involving Human Subjects and was approved by the Ethical committee of the University of Calabria (protocol number 8104). The participants provided their informed consent to participate in this study.

## Author Contributions

VB, SP, MP, and CS designed the study and contributed to the acquisition of data and had full access to all the data in the study and take responsibility for the integrity of the data and the accuracy of the data analyses. PD and CS analyzed the data. VB, AW, SP, MP, PD, and CS interpreted the data. VB and AW drafted the manuscript. SP, MP, and CS critically revised the manuscript. All authors have read the manuscript and have agreed with its submission.

## Conflict of Interest

The authors declare that the research was conducted in the absence of any commercial or financial relationships that could be construed as a potential conflict of interest.

## Footnotes

1 https://www.youtube.com/watch?v=Gr0Nsrz7W3s

2 http://www.governo.it/it/coronavirus-misure-del-governo

3 https://www.youtube.com/watch?v=eBjPInpWwnM
